# High prevalence of methicillin resistant staphylococci strains isolated from surgical site infections in Kinshasa

**DOI:** 10.11604/pamj.2014.18.322.4440

**Published:** 2014-08-21

**Authors:** Jean-Marie Liesse Iyamba, José Mulwahali Wambale, Cyprien Mbundu Lukukula, Ntondo za Balega Takaisi-Kikuni

**Affiliations:** 1 Laboratory of Experimental and Pharmaceutical Microbiology, Faculty of Pharmaceutical Sciences, University of Kinshasa, Democratic Republic of Congo

**Keywords:** Methicillin resistant staphylococci, antibiotic sensitivity, surgical site infections, Kinshasa

## Abstract

**Introduction:**

Surgical site infections (SSIs) after surgery are usually caused by *Staphylococcus aureus* and coagulase-negative staphylococci (CNS). In low income countries, methicillin resistant *Staphylococcus aureus* (MRSA) and methicillin resistant coagulase-negative staphylococci (MR-CNS) surgical site infections are particularly associated with high treatment cost and remain a source of mortality and morbidity. This study aimed to determine the prevalence and the sensitivity to antibiotics of MRSA and MR-CNS isolated from SSIs.

**Methods:**

Wound swabs were collected from 130 hospitalized surgical patients in two major hospitals of Kinshasa. *S. aureus* and CNS strains were identified by standard microbiological methods and latex agglutination test (Pastorex Staph-Plus). The antibiotic susceptibility of all staphylococcal strains was carried out using disk-diffusion method.

**Results:**

Eighty nine staphylococcal strains were isolated. Out of 74 *S. aureus* and 15 CNS isolated, 47 (63.5%) and 9 (60%) were identified as MRSA and MR-CNS respectively. Among the MRSA strains, 47 strains (100%) were sensitive to imipenem, 39 strains (89%) to amoxycillin-clavulanic acid and 38 strains (81%) to vancomycin. All MR-CNS were sensitive to imipenem, amoxycillin-clavulanic acid and vancomycin. The isolated MRSA and MR-CNS strains showed multidrug resistance. They were both resistant to ampicillin, cotrimoxazole, erythromycin, clindamycin, ciprofloxacin, cefotaxime and ceftazidime.

**Conclusion:**

The results of the present study showed a high prevalence of MRSA and MR-CNS. Imipenem, amoxycillin-clavulanic acid and vancomycin were the most active antibiotics. This study suggests that antibiotic surveillance policy should become national priority as MRSA and MR-CNS were found to be multidrug resistant.

## Introduction

Patients that are undergoing surgery or surgical procedures are at risk of acquiring infections at the site of incision. Surgical site infections (SSIs) are defined as infections occurring up to 30 days after surgery (or up to one year after surgery in patients receiving implants) and affecting either the incision or deep tissue at the operation site [1]. Despite improvements in prevention, SSIs remain a significant clinical problem as they are associated with substantial mortality and morbidity and impose severe demands on healthcare resources [2]. Microbiology studies on SSIs have shown that most of them are caused by skin-derived bacteria such as *Staphylococcus aureus* and coagulase-negative staphylococci [2, 3]. These bacteria, particularly methicillin resistant *Staphylococcus aureus* (MRSA) and methicillin resistant-coagulase negative staphylococci (MR-CNS) are known for they ability to develop resistance to multiple antibiotics [4, 5]. Studies on antimicrobial resistance of staphylococci and other bacteria isolated from hospitalized surgical patients were conducted in some African countries [6–9]. But in the Democratic Republic of Congo (DRC), the resistance pattern of *Staphylococcus*, especially MRSA and MR-CNS from SSIs to antibiotics remains unknown due to lack of antibiotic surveillance policies. The objective of the present study was to determine the prevalence and antibiotic susceptibility patterns of MRSA and MR-CNS isolated from SSIs in two major hospitals of Kinshasa.

## Methods

### Origin of staphylococci strains and laboratory procedure

This study was conducted between January to October 2013 in the Provincial General Reference Hospital of Kinshasa (HGPRK) and in Clinic of University of Kinshasa (CUK), a tertiary hospital structure. The clinical samples were collected for diagnostic purposes by the bacteriology laboratories of these hospitals and were from purulent lesions of infected sites of 130 hospitalized surgical patients. Infected sites were aseptically cleaned using normal saline and sterile gauzes. Then a wound swab from each patient was collected using sterile cotton swabs in Stuart transport media (Difco, BD Franklin Lakes, NJ, USA) and was processed in the microbiology laboratory of the Faculty of Pharmaceutical Sciences, University of Kinshasa.

### Isolation and Identification of staphylococci

Wound swabs were inoculated into mannitol-salt agar (Liofilchen, Roseto degli Abbruzzi, Italy) and incubated at 37°C for 24 hours. The genera *Staphylococcus* was confirmed using Gram staining and catalase reaction. The identification of *Staphylococcus aureus* strains was performed with latex agglutination test (Pastorex Staph-Plus, BioRad, Marnes-la-Coquette, France) and DNase test. All staphylococcal strains, negative for latex agglutination and DNase tests, were considered as coagulase negative staphylococci.

### Antibiotic susceptibility tests

Antibiograms of each isolated strains using the diffusion method on Mueller Hinton Agar were realized with the following antibiotic disks (Liofilchen, Roseto degli Abruzzi, Italy): amoxicillin-clavulanic acid (30 µg), ampicillin (10 µg), ciprofloxacin (5 µg), cefotaxime (30 µg), ceftazidime (30 µg), clindamycin (2 µg), erythromycin (15 µg), imipenem (10 µg), cotrimoxazole (25 µg), and vancomycin (30 µg). Test for methicillin resistance was performed with diffusion method using oxacillin (1 µg) on Mueller Hinton agar with 4% NaCl. Evaluation of the results was done according to the criteria of Clinical Laboratory Standards Institute (CLSI) [10]. *S. aureus* ATCC 25923 and *Staphylococcus epidermidis* ATCC 12228 were used for quality control.

## Results

Eighty-nine staphylococci were isolated during the period of the study. Seventy-four strains were *S. aureus* and 15 strains were CNS. The *S. aureus* and CNS strains demonstrated good sensitivity patterns to imipenem, amoxicillin-clavulanic acid, and vancomycin. However, most *S. aureus* and CNS strains were resistant to ampicillin, cotrimoxazole, ciprofloxacin, erytromycin, clindamycin and ceftazidime (Table 1, Table 2). Out of the 74 *S. aureus* strains isolated, 47 (63.5%) were MRSA (Table 3). All these strains were sensitive to imipenem, 39 strains (89%) to amoxicillin-clavulanic acid and 38 strains (81%) to vancomycin. Nine (60%) of 15 CNS strains were MR-CNS (Table 4). All MR-CNS strains were fully sensitive to amoxicillin-clavulanic acid, imipenem, and vancomycin. The MRSA and MR-CNS strains demonstrated a high level of resistance to ampicillin, cotrimoxazole, erythromycin, clindamycin, ciprofloxacin, cefotaxime and ceftazidime. Multidrug resistance was observed in MRSA and MR-CNS strains (Table 5).

**Table 1:** Antibiotic susceptibility patterns of all *S. aureus* strains

	*S. aureus* (n= 74)	
Antibiotics	Sensitive (%)	Resistant (%)
Ampicillin	16 (21.6%)	58 (78.4%)
Amoxicillin/Clav.acid	65 (88%)	9 (12%)
Ceftazidime	37 (50%)	37 (50%)
Cefotaxime	44 (59.5%)	30 (40.5%)
Ciprofloxacin	24 (32.4%)	50 (67.6%)
Clindamycin	22 (29.7%)	52 (70.3%)
Cotrimoxazole	20 (27%)	54 (73%)
Erythromycin	23 (31%)	51 (69%)
Imipenem	74 (100%)	0 (0%)
Vancomycin	63 (85%)	11 (15%)

**Table 2:** Antibiotic susceptibility patterns of all CNS strains

	CNS (n = 15)	
Antibiotics	Sensitive	Resistant
Ampicillin	6 (40%)	9 (60%)
Amoxicillin/Clav.acid	14(93.3%)	1 (7.3%)
Ceftazidime	7 (46.7%)	8 (53.3%)
Cefotaxime	6 (40%)	9 (60%)
Ciprofloxacin	1 (6.7%)	14(93.3%)
Clindamycin	2 (13.3%)	13 (86.7%)
Cotrimoxazole	5 (33.3%)	10 (66.7%)
Erythromycin	4 (26.7%)	11 (73.3%)
Imipenem	15 (100%)	0 (0%)
Vancomycin	13 (86.7%)	2 (13.3%)

**Table 3:** Antibiotic susceptibility patterns of MRSA strains

	MRSA (n = 47)	
Antibiotics	Sensitive	Resistant
Ampicillin	5(10.6%)	42 (89.4%)
Amoxicillin/Clav.acid	39 (89%)	8 (17%)
Ceftazidime	22 (42%)	25 (53%)
Cefotaxime	20 (42.6%)	27 (57.4%)
Ciprofloxacin	20 (42.6%)	27 (57.4%)
Clindamycin	15 (32%)	32 (68%)
Cotrimoxazole	11 (23.4%)	36 (76.6%)
Erythromycin	11 (23.4%)	36 (76.6%)
Imipenem	47 (100%)	0 (0%)
Vancomycin	38(81%)	9(19%)

**Table 4:** Antibiotic susceptibility patterns of MR- CNS strains

	(MR- CNS (n = 9)	
Antibiotics	Sensitive	Resistant
Ampicillin	1(11.1%)	8 (88.9%)
Amoxicillin/Clav.acid	9 (100%)	0 (0%)
Ceftazidime	5 (55.6%)	4 (44.6%)
Cefotaxime	3 (33.3%)	6 (66.7%)
Ciprofloxacin	0 (0%)	9 (100%)
Clindamycin	3 (33.3%)	6 (66.7%
Cotrimoxazole	0 (0%)	9 (100%)
Erythromycin	2 (22.2%)	7 (77.8%)
Imipenem	9 (100%)	0 (0%)
Vancomycin	9(100%)	0(0%)

**Table 5:** Different resistance patterns of MRSA and MR-CNS strains

Resistance pattern	MRSA (n= 47)	MR-CNS (n= 9)
1 AB	0 (0%)	0 (0%)
2 AB	1 (2.1%)	0 (0%)
3 AB	5 (10.6%)	0 (0%)
4 AB	10 (21.2%)	3 (33.3%)
5 AB	10 (21.2%)	2 (22.2%)
6 AB	6 (12.7%)	1 (11.1%)
7 AB	7 (14.9%)	3(33.3%)
8 AB	4 (8.5%)	0 (0%)
9 AB	2 (4.2%)	0 (0%)

AB: antibiotic

## Discussion

The results obtained in the present study demonstrated high prevalence of MRSA (63.5%) and MR-CNS (60%). This prevalence is higher in comparison with other studies conducted in some African countries. Report from Uganda demonstrated that the proportion of MRSA among *S. aureus* strains isolated from SSIs was 37.5% [6]. A survey conducted in Mulago National Referral Hospital (Uganda) on the surgical inpatients showed also an MRSA prevalence of 42% [11]. However, our results are not consistent with those obtained in Bobo Dioulasso and Nairobi where very low MRSA prevalence was observed [7, 12]. Screening of MR-CNS isolates showed a high prevalence rate. Very few African studies have been focused on the prevalence of CNS SSIs [13]. However, the current results are consistent with report from Switzerland in which the MR-CNS rate increased from 20% at the admission to 47% after surgery [14]. The highest MRSA and MR-CNS prevalence observed in our study can be attributed to the high pressure use of broad spectrum antibiotics or to the long stay hospital [15, 16]. In general, MRSA is known to be transmitted mostly by health-care contacts [17, 18]. A Congolese study among nasal staphylococcal carriers reported an MRSA prevalence of 63.1%, 66.7% and 23.3% in hospitalized patients, hospital staff team and outpatients, respectively [19]. Genomic characterization studies should be conducted in order to determine different genotypes of *Staphylococus aureus* strains circulating among SSIs patients and hospital staff. The MRSA and MR-CNS strains were generally multidrug resistant with an increasing resistance to ampicillin, cotrimoxazole, erythromycin, clindamycin, ciprofloxacin, ceftazidime and cefotaxime. This observation is in line with previous studies [20, 21]. The multidrug resistance observed is probably due to the ability of MRSA and MR-CNS strains to produce biofilms. Staphylococci are known as especially good biofilm formers, which is due primarily to the production of a series of surface molecules that promote extracellular matrix formation [22]. Colonization of a biotic surface such as wound by *S. aureus* has been demonstrated *in vivo* in pigs and the biofilms formed were less susceptible to antimicrobials than the planktonic counterparts [23].

A report from Italy also showed that *S. aureus* strains isolated from SSIs and producing a biofilm were highly resistant to antibiotic treatment [24]. This observation suggests that bacteria within the wound or the surgical lesion are encased in an extracellular polymeric matrix, change their phenotype and become recalcitrant to antibiotics and to host defenses [22]. None MR-CNS strain was resistant to amoxicillin-clavulanic acid, imipenem and vancomycine. We observed in the present study rates of Vancomycin Resistant *S. aureus* (VRSA) of 15% in all *S. aureus* strains and 19% in MRSA strains. Our findings were consistent with a previous report from Libya in which MRSA collected from different clinical specimens showed a VRSA prevalence of 17%, using disc-diffusion method [25]. The increase of VRSA prevalence could be explained not only by the long use of vancomycin, but also by an infection or concurrent colonization with vancomycin resistant enterococci [26].

## Conclusion

The results of the present study indicate that amoxicillin-clavulanic acid, imipenem and vancomycine should be recommended in the treatment of SSIs involving MRSA and MR-CNS. But, considering the emergence of multidrug resistant MRSA and MR-CNS strains, antibiotic susceptibility testing should be done prior to treatment in *S. aureus* and CNS surgical site infections.
